# The Critical Role of Potassium Efflux and Nek7 in *Pasteurella multocida*-Induced NLRP3 Inflammasome Activation

**DOI:** 10.3389/fmicb.2022.849482

**Published:** 2022-03-08

**Authors:** Yu Wang, Zheng Zeng, Jinrong Ran, Lianci Peng, Xingping Wu, Chao Ye, Chunxia Dong, Yuanyi Peng, Rendong Fang

**Affiliations:** ^1^Joint International Research Laboratory of Animal Health and Animal Food Safety, College of Veterinary Medicine, Southwest University, Chongqing, China; ^2^Chongqing Animal Disease Prevention and Control Center, Chongqing, China; ^3^Immunology Research Center, Medical Research Institute, Southwest University, Chongqing, China; ^4^Chongqing Key Laboratory of Herbivore Science, Chongqing, China

**Keywords:** *Pasteurella multocida*, potassium efflux, NLRP3 inflammasome, Nek7, immunity

## Abstract

*Pasteurella multocida* is a zoonotic pathogen causing respiratory infection in different animal species such as cattle, sheep, pigs, chickens and humans. Inflammasome is a complex assembled by multiple proteins in the cytoplasm and plays an important role in the host defense against microbial infection. Bovine *Pasteurella multocida* type A (PmCQ2) infection induces NLRP3 inflammasome activation and IL-1β secretion, but the mechanism of PmCQ2-induced activation of NLRP3 inflammasome is still unknown. Therefore, the underlying mechanism was investigated in this study. The results showed that potassium efflux mediated PmCQ2-induced IL-1β secretion and blocking potassium efflux attenuated PmCQ2-induced caspase-1 activation and ASC oligomerization. Furthermore, NIMA-related kinase 7 (Nek7) was also involved in PmCQ2-induced caspase-1 activation and IL-1β secretion. In addition, PmCQ2 infection promoted Nek7-NLRP3 interaction, which is dependent on potassium efflux. In conclusion, our results indicate the critical role of potassium efflux and Nek7 in *Pasteurella multocida*-induced NLRP3 inflammasome activation, which provides useful information about *Pasteurella multocida-*induced host immune response.

## Introduction

*Pasteurella multocida* (*P. multocida*) is a conditional pathogen that causes a variety of diseases and closely related to the outbreak of animal diseases (Kubatzky, 2012; Wilson and Ho, 2013; Guan et al., 2020). So far, *P. multocida* is identified and classified as five capsular serogroups (serogroups A, B, D, E and F) (Townsend et al., 2001). These serotypes of *P. multocida* causes severe infections with different clinical symptoms in different animals. For example, *P. multocida* of B and E serotypes infect cloven-hoofed animals including cattle and sheep, causing hemorrhagic septicemia. *P. multocida* of serotype A and F infection cause avian cholera. *P. multocida* of serotype D (a small amount of type A) cause infection in pigs or rabbits, leading to atrophic rhinitis (Wilkie et al., 2012). Out of five serogroups, type A *P. multocida* is one of the main pathogens of bovine respiratory syndrome leading to high morbidity and mortality, which brings big economic loss in bovine industry. So far, there is no efficient therapies to prevent or treat its infection. Therefore, it is necessary to investigate the interaction of *P. multocida* and the host, which will contribute to the development of novelty therapeutics from the host perspective.

The study on genomic analysis of a highly virulent *P. multocida* strain PmCQ2 induced host response suggested that PmCQ2 infection may activate NOD-like receptor signaling pathway (Wu et al., 2017; Li et al., 2020; He et al., 2021). The central protein of the inflammasome complex include NOD-like receptor (NLR) family, AIM2-like receptor (ALR) family, and pyrin protein. Assembly and activation of inflammasome also require apoptosis-associated speck like protein (ASC) to recruit caspase-1 protein. Inflammasomes play an important role in host defense against pathogen infection and are a core component of innate immune surveillance (Deets and Vance, 2021). NLR Family Pyrin Domain Containing 3 (NLRP3) inflammasome is the most studied and can be activated by different microorganisms including bacteria and viruses (Wang et al., 2018; Zhi et al., 2020; Ta and Vanaja, 2021; Ye et al., 2021). Our previous research showed that *P. multocida* induce NLRP3 inflammasome activation, leading to maturation and secretion of IL-1β *in vivo* and *in vitro* (Fang et al., 2019). However, the upstream signaling mechanism of NLRP3 inflammasome activation is still unclear.

NLRP3 inflammasome activation is involved in multiple signals, such as efflux of potassium (K^+^), lysosomal disruption, mitochondrial dysfunction and metabolic changes (Kelley et al., 2019; Swanson et al., 2019). Of these factors, K^+^ efflux is thought to be a common trigger of NLRP3 inflammasome activation. Recently, it has been reported that K^+^ efflux-mediated NLRP3 inflammasome activation requires a NLRP3-binding protein called Nek7 which is a member of the mammalian NIMA-related kinases (Neks) family (He et al., 2016). Nek7 has been identified to play an important role to activate NLRP3 inflammasome *via* direct interaction with NLRP3 protein (Liu G. et al., 2020). Although Nek7 has been identified to act as downstream of K^+^ efflux to involve in NLRP3 inflammasome activation, it is still unknown about the mechanism of Nek7-NLRP3 interaction in response to *P. multocida* infection.

Therefore, in this study, we investigated the mechanism of *P. multocida*-induced NLRP3 inflammasome activation as well as Nek7-NLRP3 interaction in macrophages. The results showed that *P. multocida* induced Nek7 binding to NLRP3 protein to regulate the activation of NLRP3 inflammasome, and then ASC was recruited to activate caspase-1, leading to the maturation and secretion of IL-1β, and this process depended on K^+^ efflux. Our research provides further understanding of the mechanism of *P. multocida*-induced pro-inflammatory immune response.

## Materials and Methods

### Mice

The wild-type (WT) C57BL/6 mice were purchased from Chongqing Academy of Chinese Material Medical (Chongqing, China). *Nlrp3^–/–^*, *Asc^–/–^* and *Casp1*/*11^–/–^* mice were kindly gifted by Dr. Feng Shao from the NIBS (National Institute of Biological Sciences, Beijing, China). All gene knockout mice were on a C57BL/6 background and maintained in Specific Pathogen Free (SPF) conditions for being used at 8-10 weeks old. All of animal experiments were approved by the Southwest University Ethics Committee, Chongqing, China (IACUC-2019-0112-02).

### Bacterial Strains

The highly virulent bovine *P. multocida* capsular type A PmCQ2 (GenBank accession number: LIUN00000000) was isolated from the lungs of calves with pneumonia in Chongqing, China (Du et al., 2016). The bacteria were stored at −80 °C and incubated on Martin’s agar plates at 37°C for 18–24 h. Then, the single colony was incubated in 5 mL Martin Broth (Solarbio, China) at 37°C for 12 h. Bacterial concentration was determined by colony counting, and the bacteria was diluted as indicated concentration for assays described below.

### Preparation of Peritoneal Macrophages and Bacterial Infection *in vitro*

Peritoneal exudate cells (PECs) were collected as previously reported (Fang et al., 2019). Briefly, mice were injected intraperitoneally with 2 mL of 4% thioglycolate (Eiken, Japan) and PECs were collected 3 days later. PECs were suspended with RPMI 1640 medium containing 10% FCS. Cells were seeded into each well at 2 × 10^5^ cells/well for 48-well plates or 1.0 × 10^6^ cells/well for 12-well plates and incubated at 37°C with 5% CO_2_. After 2 h incubation, the non-adherent cells were removed and adherent cells were infected with PmCQ2 at a multiplicity of infection (MOI) of 1 for 9 h. Then 100 μg/mL of ciprofloxacin (Solarbio, China) was added for an additional 15 h. After 24 h incubation, supernatants and cell lysates were collected for assays described below. To inhibit the outflow of potassium ions, cells were pretreated with KCl, Quinine (Sigma, United States) or Glibenclamide (dilute with DMSO, TCI, China) for 30 min before bacterial infection.

### Enzyme Linked Immunosorbent Assay

Cells were prepared in 48-well plates and infected with PmCQ2 as described above. After infection, supernatants were collected and Enzyme Linked Immunosorbent Assay (ELISA) was used to measure the levels of cytokines according to the manufacturers’ instructions. The kits of IL-1β, TNF-α, and IL-6 were purchased from Invitrogen (CA, United States).

### Western Blot Analysis

Cells were prepared in 12-well plates and infected with PmCQ2 as described above. After infection, supernatants were collected and concentrated using 20% (w/v) trichloroacetate, and the cells were lysed with 1 × SDS Loading buffer (Beyotime, China). Cell lysates were subjected to 12% SDS-PAGE and subsequently transferred onto a polyvinylidene difluoride (PVDF) membrane by electroblotting. The membranes were blocked with 5% non-fat dry milk and then immunoblotted with indicated antibodies (Abs) including anti-IL-1β Ab (R&D, United States), anti-Caspase1-p20 Ab (AdipoGen, United States), anti-ASC Ab (Cell signaling technology, Danvers, MA, United States), anti-Nek7 Ab (Abcam, Cambridge, UK), anti-NLRP3 Ab (Wanlei Life Sciences, Shenyang, China) and anti-GAPDH Ab (Beyotime, Beijing, China). Finally, the distinct protein bands were detected by ECL detection reagent (Biosharp, China).

### ASC Oligomerization

Cells were prepared in 12-well plates and infected with PmCQ2 as described above. After infection, cells were lysed with cold PBS containing 0.5% Triton X-100 and cell lysates were centrifuged at 13,000 rpm for 15 min at 4°C to obtain cell pellets. Then, the pellets were washed twice with cold PBS and suspended in 200 μL PBS. Subsequently, the resuspended pellets were cross-linked with 2 mM fresh disuccinimidyl suberate (DSS) at 37°C for 30 min and the pellets were centrifuged at 13,000 rpm for 15 min at 4°C. Finally, the cross-linked pellets were redissolved in 30 μL 1 × SDS-PAGE sample loading buffer and samples were boiled for 5 min before the western blot analysis.

### RT-PCR Analysis

PECs were infected with PmCQ2 as described above. After infection, total RNA was extracted using the TRIzol Reagent (Life Technologies Carlsbad, CA, United States) according to the manufacturers’ instructions, and then cDNA was synthesized using PrimeScript^®^ RT reagent Kit (Perfect Real Time) (Takara, Japan). Subsequently, quantitative real time-PCR (RT-PCR) was performed using the CFX96 (Bio-Rad, United States). Primers were used as follows: β*-actin* forward 5′-TGGAATCCTGTGGCATCCATGAAAC and reverse 5′-TAAAACGCAGCTCAGTAACAGTCCG, *NLRP3* forward 5′-CTTTCTGGACTCTGACCGGG and reverse 5′-CTCCCATTCTGGCTCTTCCC. The relative mRNA expression was analyzed against the expression level of β-actin.

### Immunoprecipitation

After infection, cells were lysed in ice-cold cell lysis buffer (20 mM Tris, pH 7.5, 150 mM NaCl, 1% Triton X-100, and sodium pyrophosphate, β-glycerophosphate, EDTA, Na_3_VO_4_, leupeptin) (Beyotime, Beijing, China) for western blot and Immunoprecipitation (IP). Cell lysates were clarified by centrifugation (12,000 rpm) at 4°C for 10 min, and were incubated with anti-NLRP3 (1:100) or Rabbit IgG (Beyotime, Beijing, China) as negative control at 4°C overnight. The proteins bound to antibody were pulled down by protein A + G beads (Beyotime, Beijing, China) and subjected to immunoblotting analysis.

### RNA Interference

PECs were transfected using Lipofectamine 3000 (Thermo Fisher Scientific, United States) with 60 nM of Nek7 siRNA (Sangon Biotech, 5′-GAUAGACUGUGUUUAUAGATT-3′) or 60 nM of control siRNA (Sangon Biotech, 5′-UUCUCCG AACGUGUCACGUTT-3′) for 48 h before infection and then were infected with PmCQ2 as described before. Finally, cell lysates were collected for RT-PCR and western blot. The relative Nek7 gene expression levels were normalized against β-actin expression.

### Immunofluorescence Microscopy

Cells were prepared as described and infected with PmCQ2 for 3 or 4 h. After infection, cells were fixed with 4% paraformaldehyde (Sango Biotech, Shanghai, China) for 30 min at room temperature (RT) and then blocked with 5% Bovine Serum Albumin (BSA) for 1 h at RT. After washing steps, primary antibodies (anti-ASC Ab, Santa Cruz, CA; anti-NLRP3 Ab, Wanlei Life Sciences, Shenyang, China) were added and incubated overnight at 4°C. Next, Goat anti-rabbit IgG (H&L) Alexa fluor 594 (Abcam, United Kingdom) was added after washing with PBS at RT for 1 h. Subsequently, DAPI (Beyotime Biotechnology, Shanghai, China) was added and incubated in the dark for 5 min. Finally, anti-fluorescence attenuation mounting tablets (Solarbio, Beijing, China) were used and the results were observed an inverted fluorescence microscope (Olympus, Tokyo, Japan).

To show the extent of speck formation more intuitively, the percentage of cells that contained a speck was determined. Cells from five different fields (average of 100 cells/field) were counted based on DAPI-stained nuclei for each of different experiments. Images were analyzed using ImageJ. The data is expressed as the percentage of cells with specks per number of cells per field.

### Statistical Analysis

All data were presented as mean ± SD of three independent experiments and analyzed by Student’s *t*-test for two-group comparison. Statistical significance was shown as *p < 0.05, ^**^p < 0.01, and n.s means no significance. All the graphs were made by GraphPad Prism software.

## Results

### K^+^ Efflux Is Required for PmCQ2-Induced IL-1β Secretion

**FIGURE 1:**
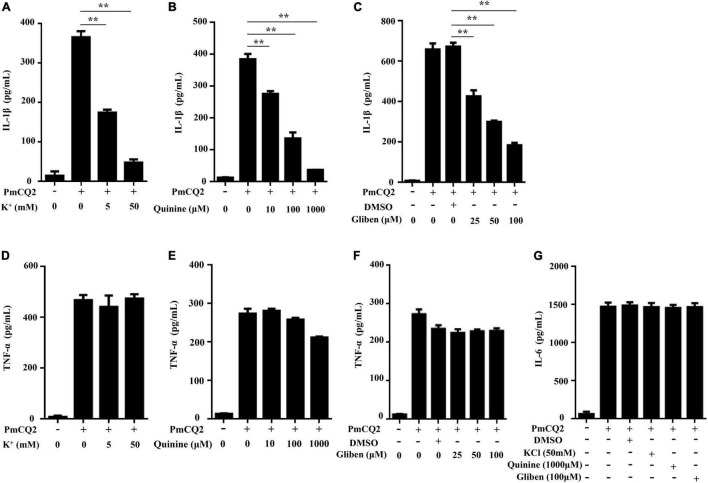
K^+^ efflux is required for PmCQ2-induced IL-1β secretion. PECs were pretreated with different inhibitors including Quinine or Glibenclamide (Gliben) or DMSO 30 min prior to PmCQ2 infection to prevent K^+^ efflux. Next, cells were uninfected (UI) or infected with PmCQ2 at a MOI of 1 in the presence or absence of KCl for 9 h and then ciprofloxacin (final concentration, 100 μg/ml) was added for an additional 15 h. After 24 h incubation, the levels of IL-1β **(A–C)**, TNF-α **(D–F)** and IL-6 **(G)** in the supernatants were determined by ELISA. Data are presented as the mean ± SD of triplicate independent experiments. Statistical significance was shown as **p < 0.01.

Our previous study showed that PmCQ2-induced IL-1β secretion was NLRP3-dependent (Fang et al., 2019), but the exact mechanism of PmCQ2-induced NLRP3 inflammasome activation remains unknown. Therefore, to explore the role of K^+^ efflux in PmCQ2-induced NLRP3 inflammasome activation, different K^+^ channel inhibitors including KCl, Quinine and Glibenclamide were used to pretreat macrophages prior to infection to inhibit potassium efflux. Our results showed that these K^+^ channel inhibitors significantly reduced PmCQ2-induced IL-1β secretion in macrophages in a concentration-dependent manner (Figures 1A–C) while the secretion of TNF-α and IL-6 were not affected (Figures 1D–G). These results suggest that potassium efflux is essential for IL-1β secretion in PmCQ2-infected macrophages.

### K^+^ Efflux Involves in PmCQ2-Induced Caspase-1 Activation and ASC Oligomerization

**FIGURE 2:**
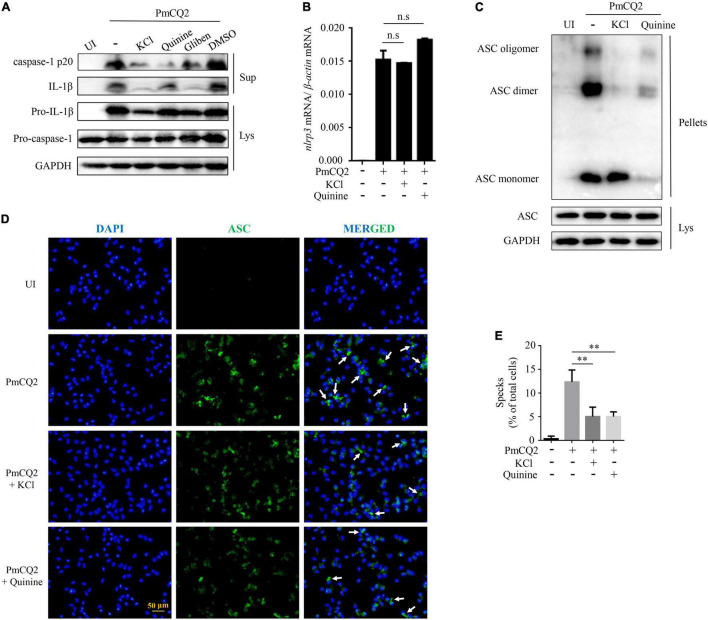
K^+^ efflux involves in PmCQ2-induced caspase-1 activation and ASC oligomerization. PECs were pretreated with 1,000 μM Quinine, 100 μM Glibenclamide and 100 μM DMSO 30 min prior to PmCQ2 infection. Next, cells were uninfected (UI) or infected with PmCQ2 at a MOI of 1 in the presence or absence of 50 mM KCl for 9 h and then ciprofloxacin (final concentration, 100 μg/ml) was added for an additional 15 h. After 24 h incubation, supernatants and cell lysates were collected. The formation of caspase-1 (p20: subunit; p45: precursor) and IL-1β (p17: subunit; p31: precursor) were detected by western blot analysis **(A)**. The level of NLRP3 mRNA expression was analyzed by RT-PCR **(B)**. The formation of ASC oligomerization was detected by immunoblotting **(C)**. Representative images of ASC speck are shown **(D)** by immunofluorescent staining (white arrows, ASC speck) **(D)**. The number of specks in PmCQ2-Infected WT Macrophages was quantified and expressed as the percentage of specks per cell number. The percentage of cells with specks relative to the total number of cells is shown **(E)**. Statistical significance was shown as **p < 0.01, no significance was shown as n.s.

Our previous study has shown that PmCQ2-induced IL-1β secretion in macrophages was caused by NLRP3 inflammasome assembly to activate caspase-1 activity. To identify the role of K^+^ efflux in PmCQ2-induced NLRP3 inflammasome assembly, caspase-1 activation and the formation of ASC oligomerization were investigated. Our results showed that the inhibition of K^+^ efflux significantly reduced PmCQ2-induced IL-1β secretion and caspase-1 (p20) maturation (Figure 2A). Notably, PmCQ2-induced NLRP3 transcription was not affected by inhibited K^+^ channel in macrophages (Figure 2B). Similarly, the expression of adaptor protein ASC as the important component of NLRP3 inflammasome was not regulated by K^+^ efflux (Figure 2C). However, the formation of ASC dimer and oligomer, which is critical for NLRP3 activation, were significantly reduced *via* inhibition of K^+^ efflux in PmCQ2-infected macrophages (Figure 2C). Interestingly, Quinine seems to be different with KCl, Quinine exerts an inhibitory effect on ASC monomer after ASC cross-linking but has no effect on ASC in lysate. Similarly, the results of immunofluorescence also showed that blocking K^+^ efflux significantly decreased the formation of ASC specks (Figure 2D, white arrows). In addition, we analyzed the percentage of cells (compared to the total number of cells) with ASC specks, the treatment of PmCQ2-infected cells with inhibitors showed a significant reduction in ASC specks (Figure 2E). These results suggest that K^+^ efflux is a necessary upstream signaling of PmCQ2-induced NLRP3 inflammasome activation, which induces ASC oligomerization and caspase-1 activation, leading to IL-1β.

### Nek7 Is Essential for PmCQ2-Induced Formation of ASC Specks and Maturation of Caspase-1 and IL-1β

**FIGURE 3:**
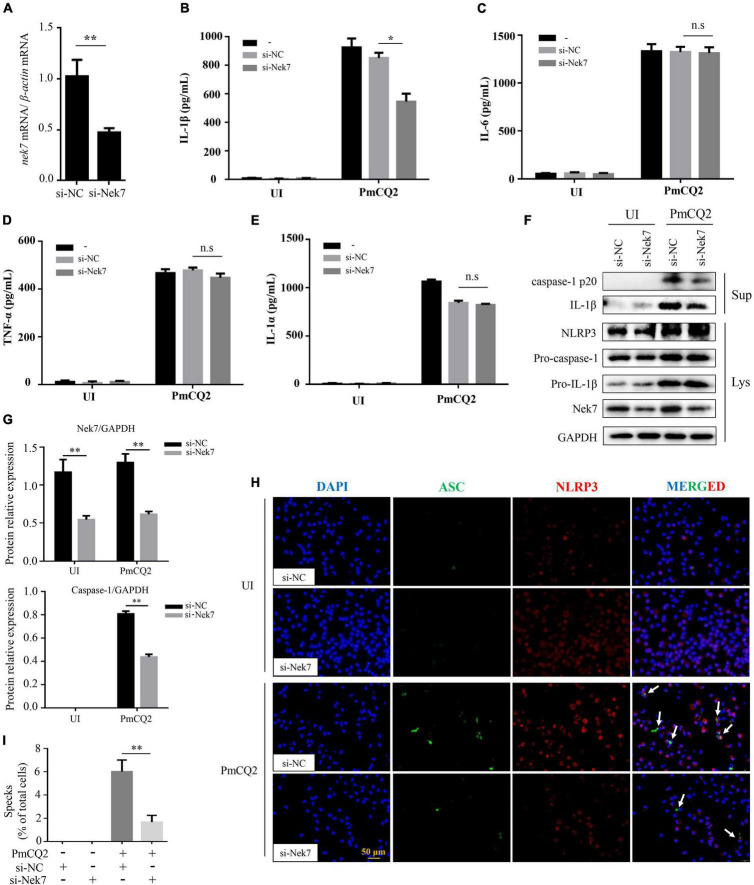
Nek7 is essential for PmCQ2-induced formation of ASC specks and maturation of caspase-1 and IL-1β. Nek7 knockdown in macrophages was performed using Nek7 siRNA transfection as confirmed for 48 h by RT-PCR, immunoblotting and immunofluorescence and then cells were infected as described above. Nek7 mRNA expression level was determined by RT-PCR **(A)**. After 48 h of si-RNA transfection, PECs were infected with PmCQ2 for 9 h and then ciprofloxacin (100 μg/ml) was added for an additional 15 h. After 24 h incubation, the secretion levels of IL-1β **(B)**, IL-6 **(C)**, TNF-α **(D)**, and IL-1α **(E)** in si-NC transfected or si-Nek7 transfected cells in uninfected and infected cells were detected by ELISA. The protein levels of NLRP3, IL-1β (p31, p17), and caspase-1 (p45, p20) were detected by immunoblotting **(F)**. Relative Nek7 or caspase-1 protein expression level against GAPDH is quantified **(G)**. The formation of ASC specks and combination of ASC and NLRP3 were detected by immunofluorescent staining Representative images of ASC speck and NLRP3 are shown **(H)** by immunofluorescent staining (white arrows, ASC speck). The number of specks in WT macrophages transfected with si-NC or si-Nek7 was quantified and expressed as a percentage of the number of specks per cell. The percentage of cells with specks relative to the total number of cells is shown **(I)**. Statistical significance was shown as *p < 0.05, **p < 0.01, no significance was shown as n.s.

It has been discovered that Nek7 acts on the downstream of K^+^ outflow, directly binds to NLRP3 and regulates NLRP3 activation. To investigate whether Nek7 mediates PmCQ2-induced NLRP3 inflammasome activation, resulting in IL-1β secretion in macrophages, Nek7 expression was genetically modified in primary macrophages using siRNA knock-out. The results showed that the transcription level of Nek7 in macrophages after Nek7 siRNA transfection was significantly lower than that of negative control siRNA transfection (Figure 3A) and these Nek7 knock-down cells also showed decreased Nek7 protein expression by western blot analysis (Figures 3F,G), indicating the silence of *nek7* gene. Furthermore, knockdown of Nek7 significantly inhibited the secretion of IL-1β (Figure 3B), but the secretion of IL-6, TNF-α, and IL-1α were not affected during PmCQ2 infection (Figures 3C–E). Similarly, PmCQ2-induced protein expression of caspase-1 and IL-1β were also attenuated in si-Nek7 cells while the NLRP3 protein expression was not affected (Figures 3F,G). In addition, the results of immunofluorescence also showed the formation of ASC specks and the combination of ASC and NLRP3 was significantly reduced after Nek7 knockdown (Figure 3H). Similarly, the percentage of cells with ASC specks after transfection is also consistent with this result (Figure 3I). These results demonstrate the involvement of Nek7 in NLRP3 inflammasome activation in response to PmCQ2 infection.

### K^+^ Efflux Mediates PmCQ2-Induced Nek7-NLRP3 Interaction

**FIGURE 4:**
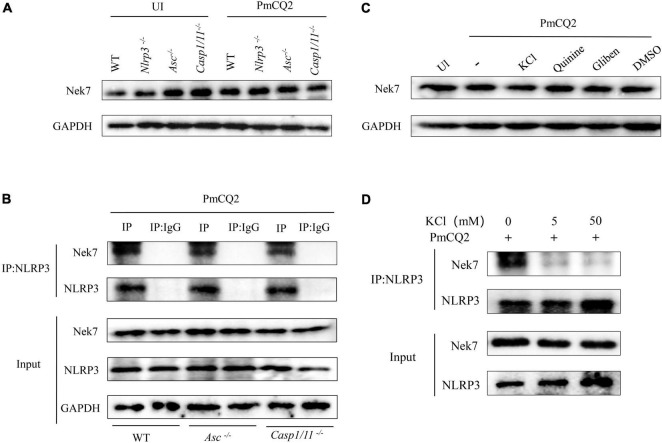
K^+^ efflux mediates PmCQ2-induced Nek7-NLRP3 interaction. PECs from C57BL/6 WT, *Casp1^–/–^*, *Asc^–/–^*, and *Nlrp3^–/–^* mice were uninfected (UI) or infected with PmCQ2 as described above. After 24 h incubation, the levels of Nek7 in cell lysates were measured by western blot **(A)**. NLRP3 protein complexes were immunoprecipitated with anti-NLRP3 antibody and analyzed by immunoblotting. The Nek7-NLRP3 interaction was detected by immunoprecipitation and immunoblotting **(B)**. Cells from WT mice were pretreated with Quinine or Glibenclamide and infected with PmCQ2. Nek7 protein expression **(C)** and Nek7-NLRP3 interaction **(D)** were detected by western blotting as well as immunoprecipitation and immunoblotting, respectively.

Nek7 plays an important role in the regulation of NLRP3 inflammasome activation as shown in Figure 3. Next, Nek7-NLRP3 interaction induced by PmCQ2 infection and the role of K^+^ efflux in this process were further studied. The results of western blot clearly showed Nek7 protein expression in infected and non-infected macrophages including *Nlrp3^–/–^*, *Asc^–/–^*, *Casp1/11^–/–^* cells (Figure 4A), suggesting that these components of inflammasome are not involved in Nek7 expression. Interestingly, PmCQ2 promoted Nek7-NLRP3 interaction by the detection of Co-IP not only in WT macrophages but also in *Asc^–/–^* and *Casp1/11^–/–^* cells (Figure 4B), demonstrating that inflammasome components are not associated with Nek7-NLRP3 interaction. Finally, to understand the mechanism of PmCQ2-induced Nek7-NLRP3 interaction, inhibitors of potassium ions were used to disrupt K^+^ efflux to investigate its role in Nek7-NLRP3 interaction. Our results showed that PmCQ2-induced combination of Nek7 and NLRP3 was abrogated in KCl-treated macrophages while Nek7 protein expression was not affected (Figures 4C,D). These results indicate that Nek7 just binds to NLRP3 protein to regulate inflammasome in response to PmCQ2 infection and this process is mediated by K^+^ efflux.

## Discussion

Bovine *P. multocida* is an important pathogen that causes bovine hemorrhagic sepsis and bovine respiratory syndrome, which has brought huge economic losses to beef cattle breeding (Turni et al., 2016). So far, vaccination and antibiotics are used to prevent and treat *P. multocida* infection. However, the efficacy of vaccine is limited in antigenic variation while the use of antibiotics is limited by the development of antibiotic resistance (Rérat et al., 2012). Therefore, the novel therapeutics against *P. multocida* are needed and the study on *P. multocida*-induced host immune response will contribute to the development of new therapeutics. Furthermore, a successful immune response is critical for efficient clearance of pathogens. Our previous study has shown that *P. multocida* activates NLRP3 inflammasome, subsequently induces caspase-1 activation which process pro-IL-1β into biologically active IL-1β, leading to IL-1β secretion and inflammatory response against infection. In this study, we further investigated the mechanism of *P. multocida*-induced NLRP3 inflammasome activation.

It has been shown that K^+^ efflux is an important factor to manipulate the activation of NLRP3 inflammasome (Zhao et al., 2020). Franchi et al. (2007) reported that intracellular concentration of K^+^ mediates NLRP3 inflammasome activation triggered by ATP and bacterial infection. Depletion of intracellular K^+^ promotes pathogens-induced caspase-1 activation, leading to maturation and secretion of IL-1β. Besides bacterial infection, Hou et al. (2021) found that bacterial toxin-stimulated NLRP3 inflammasome activation is also dependent on K^+^ efflux. Recently, it has been identified that TWIK2 expressed in the plasma membrane as a K^+^ efflux channel regulates cellular immunity, which is responsible for ATP-induced NLRP3 inflammasome activation and is sensitive to quinine (Di et al., 2018). Similarly, Glibenclamide has been also reported to attenuate the activation of NLRP3 inflammasomes by blocking K^+^ efflux (Liu et al., 2017; Shao et al., 2020). Furthermore, Liu H. et al. (2020) used Glibenclamide to block K^+^ efflux to further study the role of K^+^ efflux in Nek7-NLRP3 interaction. Therefore, in our study, quinine and Glibenclamide are used to block K^+^ efflux. Our data showed that IL-1β secretion induced by *P. multocida* was dramatically inhibited after the outflow of intracellular K^+^ was disturbed in macrophages by potassium channel blockers. Furthermore, *P. multocida*-induced both caspase-1 activation and ASC oligomerization were also attenuated by blocking intracellular K^+^ efflux. These results suggest that *P. multocida* induces K^+^ efflux and then drives NLRP3 inflammasome activation, indicating that K^+^ efflux is a common step to trigger NLRP3 inflammasome activation induced by pathogen-associated molecular patterns (PAMPs) or danger-associated molecular patterns (DAMPs).

Nek7 is a highly conserved serine/threonine kinase that is essential for the initiation of mitosis, cell cycle progression, cell division, and mitosis (Shi et al., 2016). Recently, it has been found that Nek7 is an important mediator of NLRP3 activation and being able to bind to NLRP3 protein to activate inflammasome. Chen et al. (2019) have shown that Nek7 knockdown abolishes LPS + ATP-induced caspape-1 activation and Nek7 exerts its function *via* interaction with NLRP3. Detailly, Nek7 deficiency abrogates NLRP3 oligomerization and ASC speck formation in LPS-primed macrophages (He et al., 2016). Similarly, our data showed that Nek7 knockdown significantly attenuated *P. multocida*-induced the formation of ASC specks and caspase-1 activation as well as IL-1β secretion. These results indicate that Nek7 is indispensable for *P. multocida*-induced NLRP3 inflammasome activation.

Bacterial infections have rarely been reported to activate NLRP3 inflammasome *via* Nek7-NLRP3 interaction, but researchers are gradually paying more attention on it. Recently, *Listeria monocytogenes* have been shown to promote Nek7-NLRP3 interaction through the Mst1/2-ALK pathway to activate inflammasomes and mediate cell apoptosis (Gao et al., 2021). Similarly, the activation of NLRP3 inflammasomes triggered by the *Escherichia coli* RhoGTPase-activating toxin CNF1 is also mediated by the interaction of Nek7-NLRP3 *via* K^+^ efflux (Dufies et al., 2021). In addition, the latest research shows that *Staphylococcus aureus* infection activates NLRP3 inflammasome through Nek7 and K^+^ efflux signaling (Liu et al., 2021). Our study showed that *P. multocida*-induced combination of NLRP3 and Nek7 was dependent on K^+^ efflux, which is also consistent with the findings that K^+^ efflux mediates Nek7-NLRP3 interaction (Lamkanfi et al., 2009; Di et al., 2018). Interestingly, knockout of inflammasome components including caspase-1/11 and ASC did not affect *P. multocida*-induced Nek7-NLRP3 interaction, indicating that Nek7 only binds to NLRP3 protein to activate inflammasome and this process is independent on inflammasome components. These results demonstrate the important role of K^+^ efflux and Nek7 in *P. multocida*-induced NLRP3 inflammasome activation. Although the involvement of Nek7 in inflammasome activation, it is still unclear whether other kinases or other signaling molecules are involved and therefore further research is still needed.

In summary, our study showed that *P. multocida* promotes Nek7-NLRP3 interaction to activate NLRP3 inflammasome, leading to caspase-1 activation and IL-1β secretion. In addition, K^+^ efflux acts as upstream signaling of Nek7 to mediate Nek7-NLRP3 interaction. This study provides a new insight on *P. multocida*-induced NLRP3 inflammasome activation.

## Data Availability Statement

The original contributions presented in the study are included in the article/supplementary material, further inquiries can be directed to the corresponding author/s.

## Ethics Statement

The animal study was reviewed and approved by the Southwest University Ethics Committee, Chongqing, China (IACUC-2019-0112-02).

## Author Contributions

YW, ZZ, JR, and XW performed the experiments. LP, CY, and CD helped to analyze the data. RF and YP supervised the study and designed the experiments. YW, ZZ, LP, and RF drafted the manuscript. All authors have read and agreed to the published version of the manuscript.

## Conflict of Interest

The authors declare that the research was conducted in the absence of any commercial or financial relationships that could be construed as a potential conflict of interest.

## Publisher’s Note

All claims expressed in this article are solely those of the authors and do not necessarily represent those of their affiliated organizations, or those of the publisher, the editors and the reviewers. Any product that may be evaluated in this article, or claim that may be made by its manufacturer, is not guaranteed or endorsed by the publisher.
